# Supplementary material for: Why East Asian monsoon anomalies are more robust in post El Niño than in post La Niña summers
Source: Nat Commun. 2024 Aug 27;15:7401. doi: 10.1038/s41467-024-51885-7 (PMC11349765; doi:10.1038/s41467-024-51885-7)
Supplement: Supplementary file 1 — Supplementary Information [file 41467_2024_51885_MOESM1_ESM.pdf]

Supplementary Information for

Why East Asian Monsoon Anomalies Are More Robust in Post El Niño than in Post La Niña Summers

Pengcheng Zhang, Shang-Ping Xie*, Yu Kosaka, Nicholas J. Lutsko, Yuko M. Okumura, and Ayumu Miyamoto

*Corresponding author. Email: sxie@ucsd.edu

This file includes:

Figs. S1 to S11

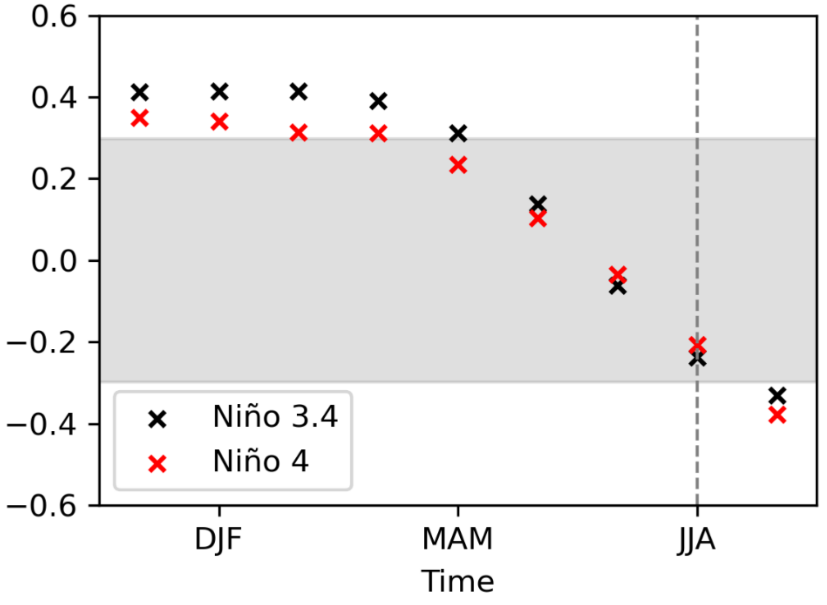

Fig. S1. Lagged correlation between the AAC index in JJA and different seasonal-mean SST indices. Black and red crosses indicate SST anomalies averaged in the Niño 3.4 and Niño 4 regions, respectively. The vertical dashed line indicates lag-0 (JJA). The gray shading denotes the 95% significance threshold based on the Student's t test.

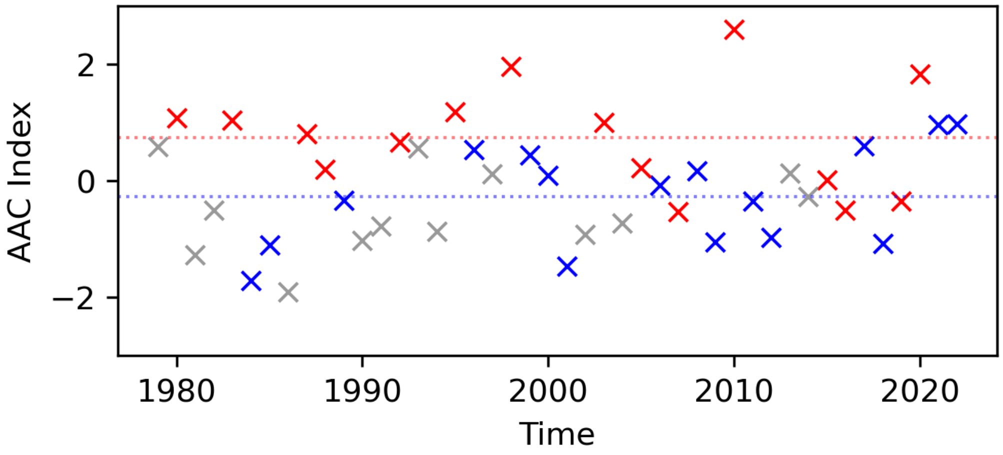

Fig. S2. Time series of the JJA AAC index. Red and blue crosses indicate post-El Niño and post-La Niña summers, respectively, in which the Niño 3.4 SST anomalies in preceding November-January are above 0.5 or below -0.5K. The colored dotted lines indicate the average AAC index in the two respective categories. The gray crosses indicate the AAC after a neutral winter.

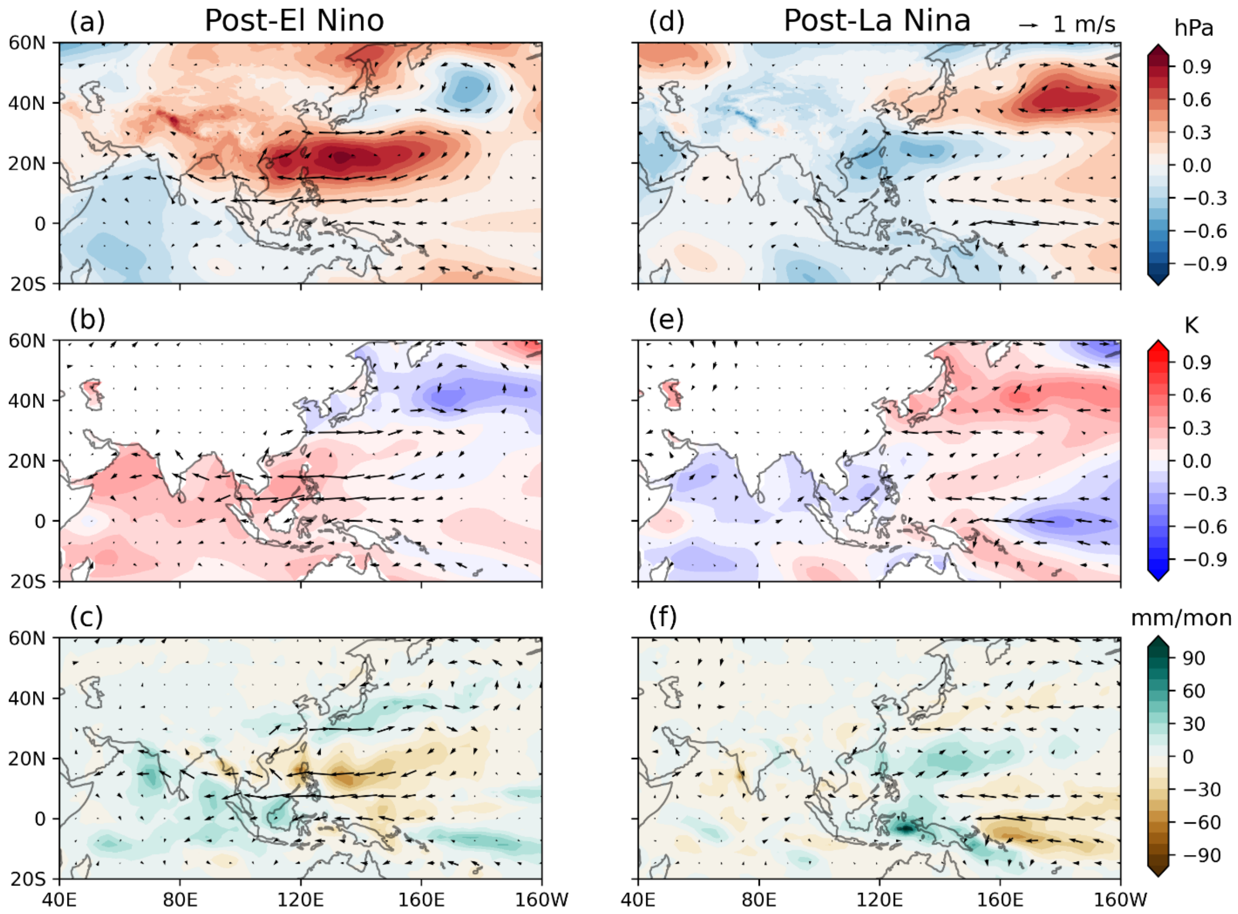

Fig. S3. Composites of observed post-ENSO summer anomalies. (**a** to **c**) SLP, SST, precipitation (shading in three panels respectively), and 850 hPa winds (arrows in all panels) averaged in post-El Niño summers. (**d** to **f**) Same as (**a** to **c**) but for post-La Niña summers. The two composites contain 15 and 16 individual cases respectively.

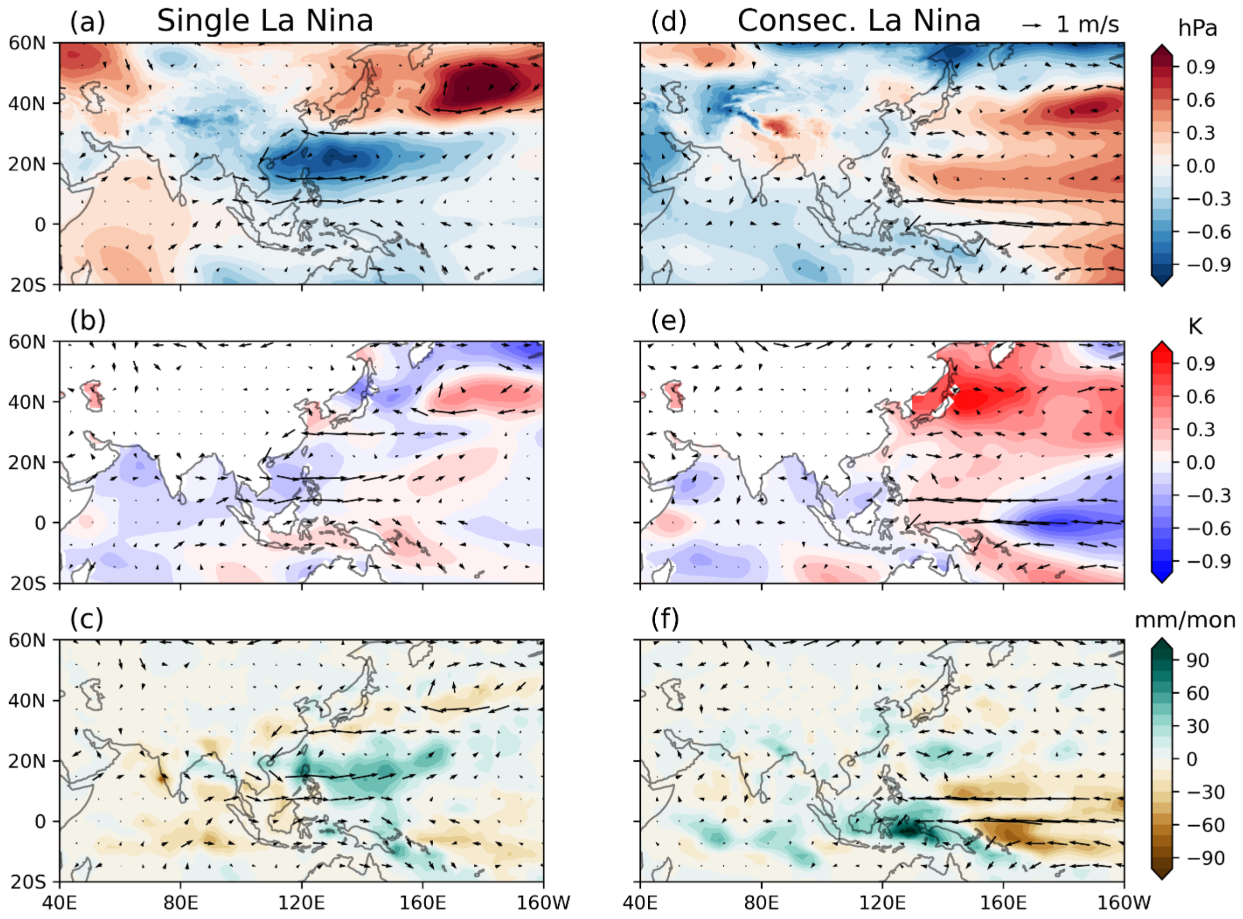

Fig. S4. Composites of observed post-La Niña summer anomalies. (**a** to **c**) SLP, SST, precipitation (shading in three panels respectively), and 850 hPa winds (arrows in all panels) averaged in post single-year La Niña summers. (**d** to **f**) Same as (**a** to **c**) but for summers between consecutive La Niña events. The two composites each contain 8 individual cases.

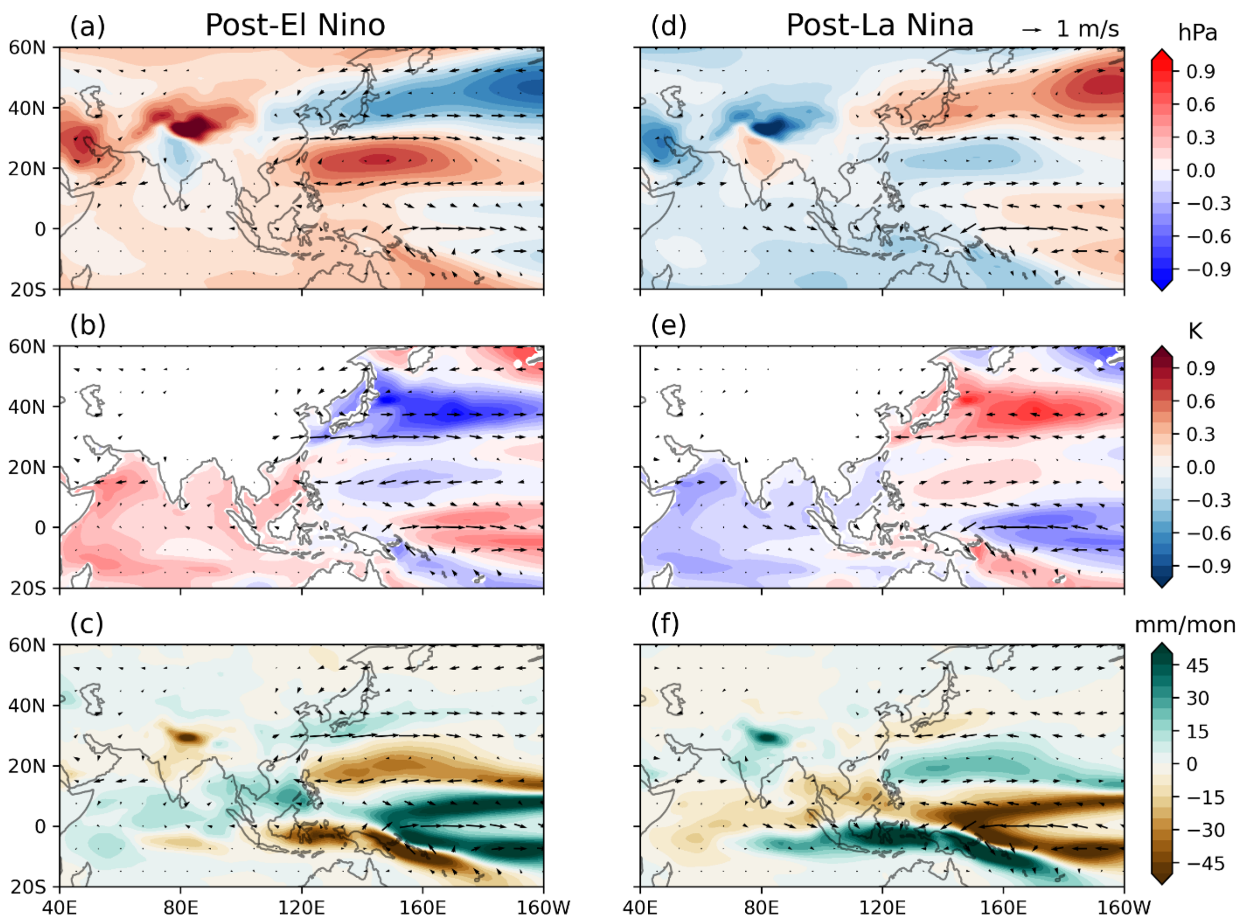

Fig. S5. Composites of CGCM simulated post-ENSO summer anomalies. (**a** to **c**) SLP, SST, precipitation (shading in three panels respectively), and 850 hPa winds (arrows in all panels) averaged in post-El Niño summers. (**d** to **f**) Same as (**a** to **c**) but for post-La Niña summers. The two composites contain 279 and 311 individual cases respectively.

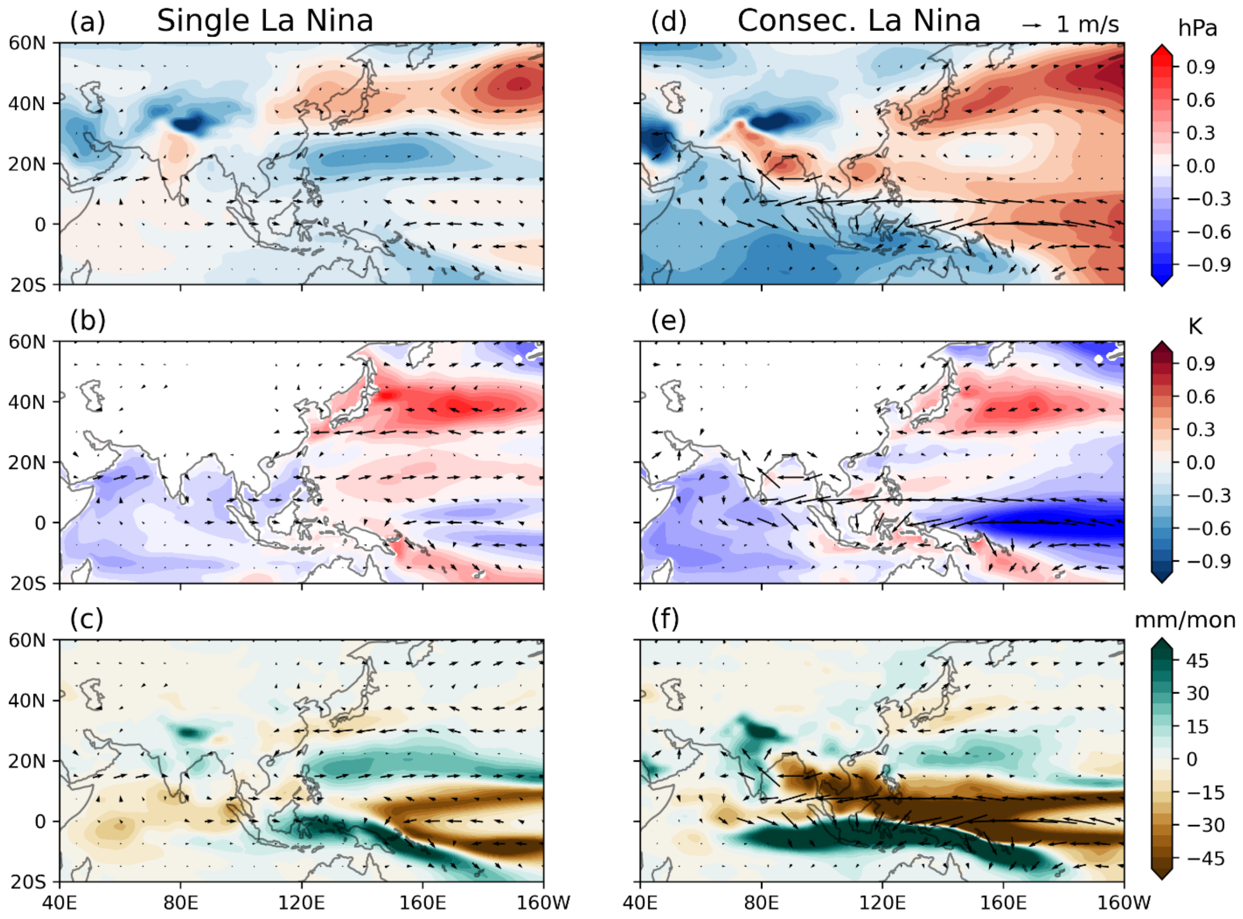

Fig. S6. Composites of CGCM simulated post-La Niña summer anomalies. (**a** to **c**) SLP, SST, precipitation (shading in three panels respectively), and 850 hPa winds (arrows in all panels) averaged in post single La Niña summers. (**d** to **f**) Same as (**a** to **c**) but for summers between consecutive La Niña events. The two composites contain 216 and 95 individual cases respectively.

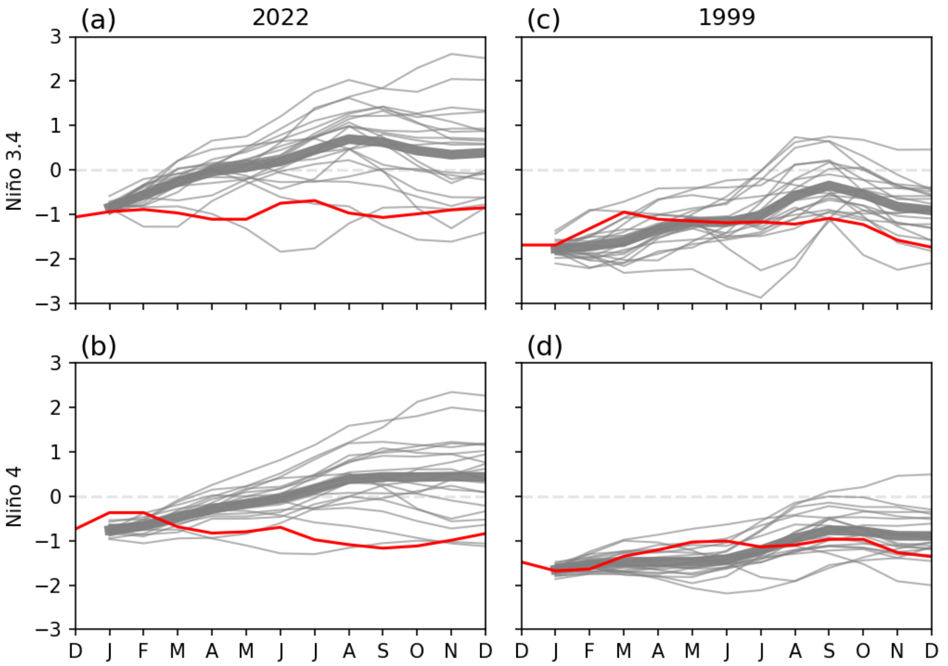

Fig. S7. Simulated ENSO evolutions by CGCM. (**a**) Initialized on Jan 1, 2022; the thin and thick grey curves indicate monthly Niño 3.4 SST anomalies (ºC) in individual ensemble members and in the ensemble mean, respectively. The red curve shows the observed values obtained from ERSST dataset. (**b**) Same as (**a**) but for Niño 4 SST anomalies. (**c**) and (**d**) Same as (**a**) and (**b**) but initialized on Jan 1, 1999.

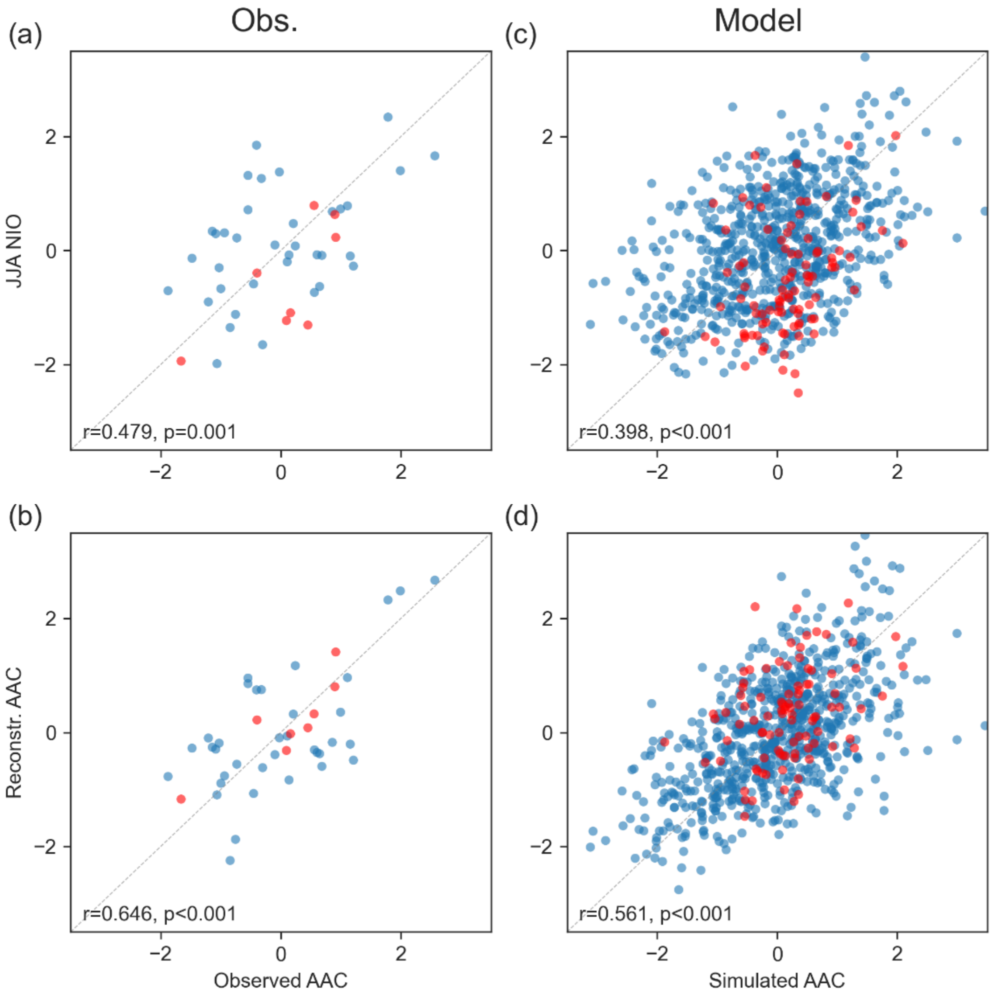

Fig. S8. As in Fig. 2 but replacing NDJ Nino 3.4 SST with JJA NIO SST. (**a**) Observed AAC index (x-axis) versus JJA NIO SST anomalies (y-axis). (**b**) Observed AAC index (x-axis) versus reconstructed AAC by JJA NIO SST and concurrent summer ENSO states (y-axis). (**c**) and (**d**) Same as (**a**) and (**b**) but for all individual ensemble members in model simulations. All the indices are normalized. The red dots denote summers during consecutive La Niña.

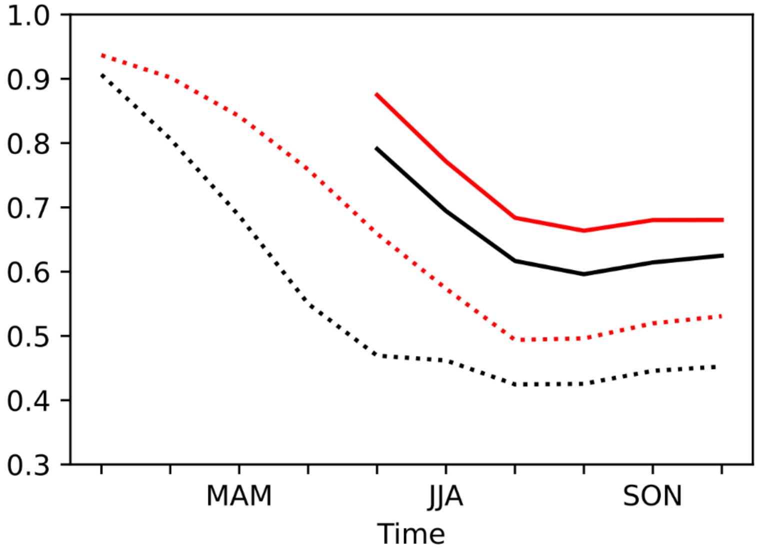

Fig. S9. ENSO Prediction skills of the ensemble mean of initialized CGCM simulations. Dotted: initialized on January 1st; Solid: initialized on May 1st. Black and red curves represent the prediction skill for Niño 3.4 and Niño 4 SST anomalies, respectively.

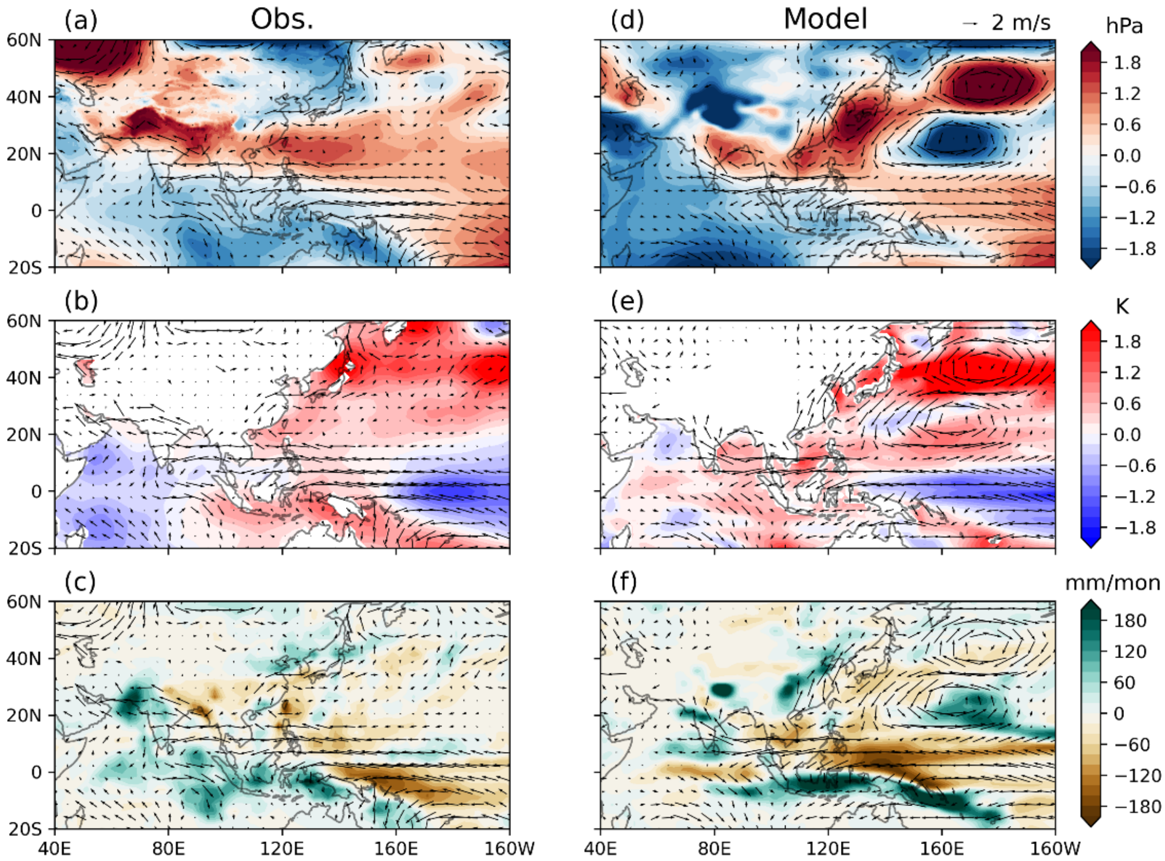

Fig. S10. Climate conditions in summer of 2022. (**a** to **c**) Observed SLP, SST, precipitation (shadings in three panels respectively), and 850 hPa winds (arrows in all panels) anomalies averaged in JJA of 2022. (**d** to **f**) Same as (**a** to **c**), but for model simulation of ensemble member #4, in which the JJA Niño 4 SST anomaly reaches -1.25 K.

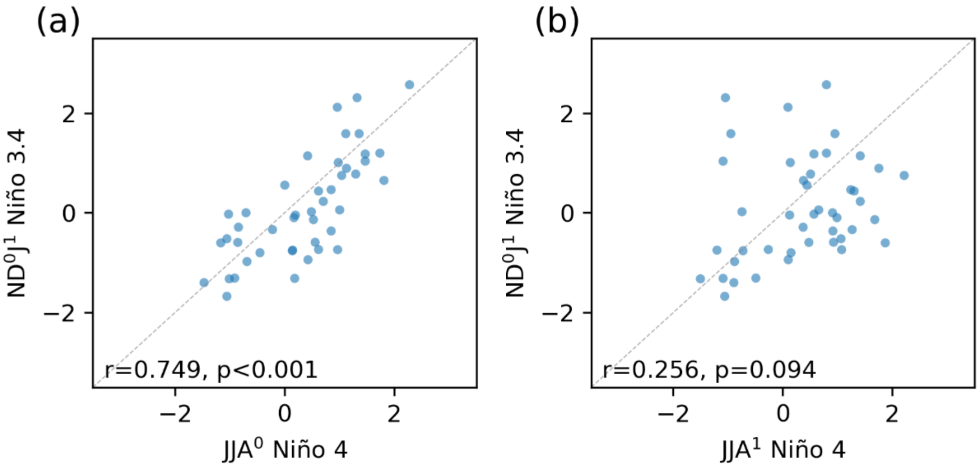

Fig. S11. Observed relationship of ENSO in different seasons. (**a**) Niño 3.4 SST anomalies in winter (NDJ) versus Niño 4 SST in the preceding summer (JJA). (**b**) Niño 3.4 SST anomalies in winter (NDJ) versus Niño 4 SST in the following summer (JJA). All the indices are normalized. The "0" and "1" superscripts indicate the year before and after the winter peak of ENSO, respectively.
